# Bacterial etiology and mortality rate in community-acquired pneumonia, healthcare-associated pneumonia and hospital-acquired pneumonia in Thai university hospital

**DOI:** 10.1038/s41598-022-12904-z

**Published:** 2022-05-30

**Authors:** Jaturon Poovieng, Boonsub Sakboonyarat, Worapong Nasomsong

**Affiliations:** 1 Department of Medicine, Phramongkutklao Hospital, Bangkok, 10400 Thailand; 2 Department of Military and Community Medicine, Phramongkutklao College of Medicine, Bangkok, 10400 Thailand; 3 Division of Infectious Disease, Department of Medicine, Phramongkutklao Hospital and College of Medicine, Bangkok, 10400 Thailand

**Keywords:** Bacteriology, Pathogens, Bacterial infection, Respiratory tract diseases

## Abstract

Pneumonia is caused by infection at the pulmonary parenchyma which constitutes a crucial risk factor for morbidity and mortality. We aimed to determine the mortality rate and its risk factors as well as etiology among inpatients with community-acquired pneumonia (CAP), hospital-acquired pneumonia (HAP) and healthcare-associated pneumonia (HCAP). A hospital-based retrospective cohort study was conducted in a university hospital located in Bangkok, Thailand. A total of 250 inpatients with pneumonia was included in the present study. The inhospital mortality rate was 1.25 (95% CI 0.99–1.56) per 100 person-days. The present study reported that overall pneumonia caused by gram-negative pathogens accounted for 60.5%. *P. aeruginosa* was a frequent gram-negative pathogen among these participants, especially among patients with HCAP and HAP. Adjusted hazard ratio (AHR) of inhospital mortality among patients with HAP was 1.75 (95% CI 1.01–3.03) times that of those among patients with CAP, while AHR for 28-day mortality among patients with HAP compared with those with CAP was 2.81 (95% CI 1.38–5.75). Individual risks factors including cardiomyopathy, active-smoker and insulin use were potential risk factors for mortality. Initial qSOFA and acid-based disturbance should be assessed to improve proper management and outcomes.

## Introduction

Pneumonia is usually caused by bacterial or viral infection at the pulmonary parenchyma which constitutes a crucial risk for morbidity and mortality worldwide^1,2^. Community-Acquired Pneumonia (CAP) is one of the most common acute infections diagnosed and treated in clinical practice^1,3,4^. In the US, the incidence of CAP in populations was 16 to 23 per 1000 person-years, and the major of those involved elderly people; moreover, patients with CAP required hospital admission approximately 5–7 per 1000 person-years^5–7^. In 2019, the Bureau of Epidemiology, Department of Disease Control in Thailand reported that the incidence of pneumonia was 388 per 100,000 populations while the mortality rate was 0.26 per 100,000 populations. However, as the incidence of pneumonia increased, the mortality rate tended to decrease when compared with that reported in the last decade^8^.

At present, pneumonia is categorized by the condition that the disease occurred to appropriate management initially. CAP is pneumonia caused by an infection outside the hospital while nosocomial pneumonia is caused by an infection after hospital admission at least 48 h and is divided in two groups, i.e., (1) hospital-acquired pneumonia (HAP) and (2) ventilator-associated pneumonia (VAP) caused by infection after at least 48 h of intubation^2^. In addition, a specific pneumonia category occurs among patients receiving care services like healthcare-associated pneumonia (HCAP), including patients hospitalized in an acute care hospital for two or more days within 90 days of the infection, residing in a long-term care facility, receiving recent intravenous antibiotic therapy, chemotherapy within the past 30 days or in the hemodialysis clinic^2^. Patients with HCAP, separated from the other pneumonia categories, may gain an advantage in the case of different causative pathogens and antimicrobial resistance profiles. However, the initial management for HCAP is currently comparable with those for CAP or HAP.

In 2019, the guidelines of the American Thoracic Society (ATS) and Infectious Diseases Society of America (IDSA) recommended that no severe CAP among adults who may have risk factors of Methicillin-resistant Staphylococcus aureus (MRSA) or *P. aeruginosa*, but without prior isolation, should be treated with CAP regimens and the HCAP entity remains unmentioned. Providing extended-spectrum antibiotic therapy instead of standard CAP regimens and obtaining the culture data will be  contributed in severe-CAP^9^. However, when the epidemiology of hospital-acquired infection indicated that it had been caused by antimicrobial resistant pathogens or *P. aeruginosa,* the extended-spectrum antibiotic therapy will be administrated for patients without severe-CAP.

In Thailand, the epidemiology of pathogens differs from that in the US in that no significant evidence exists of community MRSA, but rather, the high incidence of antimicrobial-resistant gram-negative infections^10^. Therefore, the present clinical practice guidelines of the ATS and IDSA may not be fully competent regarding clinical practices in Thailand. We hypothesized that, in CAP, HCAP and HAP, those gram-negative bacteria may differ regarding species as well as antimicrobial resistance patterns. To date, Thai clinical practice guidelines are unavailable to manage pneumonia. The investigators aimed to determine the mortality rate and epidemiology of pathogens among inpatients with CAP, HCAP and HAP in a tertiary medical center to be advantageous for appropriate treatment. Furthermore, we aimed to identify the risk factors for inhospital and 28-day mortality to improve quality of care and rational drug use.

## Methods

### Study design and subjects

A hospital-based retrospective cohort study was conducted at Phramongkutklao Hospital, a university hospital located in Bangkok, Thailand. The eligible criteria of participants comprised patients aged $\ge$ 18 years receiving a diagnosis of pneumonia and admitted at Phramongkutklao Hospital between January 1, 2015 and December 31, 2019. Pneumonia was determined according to the International Classification of Diseases, Tenth Revision codes (ICD-10) in J12-J18 presented in medical records^11^. Pneumonia was categorized in three groups including (1) CAP, (2) HCAP and (3) HAP. According to guidelines for the management of adults with pneumonia documented by the American Thoracic Society^2^, CAP was defined as pneumonia acquired outside of health care settings^3,4^ while HCAP included any patient hospitalized in an acute care hospital for two or more days within 90 days of the infection, residing in a long term care facility or a nursing home, receiving recent intravenous antibiotic therapy, chemotherapy, or wound care within the past 30 days of the current infection or attending a hospital or hemodialysis clinic^12–14^. HAP was defined as pneumonia occurring 48 h or more after admission, which was not intubated at the time of admission^14,15^. Patients receiving immunosuppressive drugs, including cytotoxic agents or a corticosteroid that was equivalent to more than 1 mg/kg/day of prednisolone for more than one month were excluded. The other exclusion criteria consisted of (1) having a history of a bone marrow or organ transplant, (2) having a history of cancer, (3) living with HIV or (4) pregnancy during admission.

### Data collection

In all, 250 inpatients with pneumonia were included in this study. Patient information was reviewed and retrieved by the internist. A standardized case report form was used to collect data of individuals from medical records, including demographic data, history of hospital admission, comorbidities, clinical signs, laboratory and radiologic findings, the process of care, microbiological etiology, and mortality outcome. The microbiological etiology data was collected based on the results of bacterial culture and polymerase chain reaction testing (if available, for respiratory virus) of respiratory specimens (sputum, tracheal aspirates, nasopharyngeal swab). According to the validation study of a practical severity assessment model for stratifying adults hospitalized with CAP and the CURB-65 score, we categorized age variables in four categories, i.e., (1) age < 65 years, (2) 65 to 74 years, (3) 75 to 84 years and (4) $\ge$ 85 years^16^. Systemic Inflammatory Response Syndrome (SIRS) was defined by the satisfaction of any two of the following criteria: (1) body temperature $>$ 38 or < 36 degrees Celsius, (2) respiratory rate > 20 breaths per minute or partial pressure of CO_2_ < 32 mmHg, (3) pulse rate > 90 beats per minute and (4) leucocyte count > 12,000 or < 4000 /microliters or over 10% immature forms or bands^17^.

The Quick Sequential (sepsis-related) Organ Failure Assessment (qSOFA) criteria, according to the Third International Consensus Definitions for Sepsis and Septic Shock included (1) respiratory rate $\ge$ 22 breaths/min, (2) altered mental status and (3) systolic blood pressure (BP) $\le$ 100 mmHg^18^. Mean arterial pressure was calculated using diastolic BP + 1/3(systolic BP-diastolic BP), and pulse pressure was calculated using systolic BP minus diastolic BP^19^.

### Ethics considerations

The study was reviewed and approved by the Institutional Review Board, Royal Thai Army Medical Department in compliance with international guidelines such as the Declaration of Helsinki, the Belmont Report, CIOMS Guidelines and the International Conference on Harmonization of Technical Requirements for Registration of Pharmaceuticals for Human Use—Good Clinical Practice (ICH-GCP), approval number R062h/63_Exp. Due to the retrospective cohort study design, a waiver of documentation of informed consent was used, and the waiver for informed consent was granted by the Institutional Review Board, Royal Thai Army Medical Department.

### Statistical analysis

All analyses were conducted using StataCorp, 2021, *Stata Statistical Software: Release 17*, College Station, TX, USA: StataCorp LLC, and IBM Corp. Released 2020. IBM SPSS Software for Windows, Version 27.0. Armonk, NY: IBM Corp. Baseline characteristics were analyzed using descriptive statistics. Categorical data were presented as percentages while mean and standard deviation (SD) were used for continuous data. The *Chi*-square test was used to compare the difference of categorical variables by pneumonia categories while continuous data were compared using ANOVA. For inhospital mortality, the person-times of observations of the participants were calculated as the duration between the date of pneumonia diagnosis and death date or date of discharge from hospital, whichever occurred first. In terms of 28-day mortality, the participants would be followed to the death date within 28 days, those patients who remained alive were right-censored on the 28^th^ day after the date of pneumonia diagnosis. The mortality rates per 100 person-days and 95% confidence interval (CI) for inhospital mortality and 28-day mortality were presented. The Kaplan–Meier estimator was used to compute survival patterns, and the log rank test was used to compare survival between pneumonia categories. Univariable and multivariable cox regression analysis were used to determine risk factors for inhospital mortality and 28-day mortality. Crude hazard ratio and adjusted hazard ratios (AHR) were presented with a 95% CI. A *p*-value less than 0.05 was considered statistically significant.

## Results

### Baseline characteristics of participants

A total of 250 inpatients with pneumonia was included in the present study. In all, 146 (58.4%) participants were males. The proportion of inpatients with CAP, HAP and HCAP was 46.4, 27.6 and 26.0%, respectively. The average age of participants with CAP, HCAP and HAP were 70.7 ± 20.0 years, 79.6 ± 11.7 years and 79.0 ± 13.6 years, respectively (*p-*value < 0.001). Of 250, 174 (69.6%) participants were married. In terms of health schemes, most participants were under the universal health coverage scheme accounting for 70.4%. The patients who needed invasive mechanical ventilation totaled 48.3, 52.2 and 66.2% among patients with CAP, HCAP and HAP, respectively (*p-*value = 0.064). Comorbidities, clinical signs and laboratory and radiologic findings of participants at baseline are presented in Table 1.

**Table 1:** Baseline characteristics of participants (N = 250).

Characteristics	Total	CAP	HCAP	HAP	*p*-value
	n (%)	n (%)	n (%)	**n (%)**	
**Overall**	250	116	69	65	
**Male **	146 (58.4)	55 (47.4)	47 (68.1)	44 (67.7)	0.005
**Age (years)**					0.053
< 65	50 (20.0)	31 (26.7)	10 (14.5)	9 (13.8)	
65–74	40 (16.0)	19 (16.4)	12 (17.4)	9 (13.8)	
75–84	84 (33.6)	42 (36.2)	21 (30.4)	21 (32.3)	
≥ 85	76 (30.4)	24 (20.7)	26 (37.7)	26 (40.0)	
Mean ± SD	75.3 ± 17.0	70.7 ± 20.0	79.6 ± 11.7	79.0 ± 13.6	< 0.001
Median (min–max)	79.5 (20.0–100.0)	77.0 (20.0–98.0)	82 (41.0–99.0)	82 (29.0–100.0)	
**Marital status**					0.159
Married	174 (69.6)	80 (69.0)	50 (72.5)	44 (67.7)	
Single	31 (12.4)	20 (17.2)	5 (7.2)	6 (9.2)	
Widowed/divorced	45 (18.0)	16 (13.8)	14 (20.3)	15 (23.1)	
**Health scheme**					0.594
Civil Servant Medical Benefit	52 (20.8)	27 (23.3)	16 (23.2)	9 (13.8)	
Universal coverage	176 (70.4)	80 (69.0)	47 (68.1)	49 (75.4)	
Others	22 (8.8)	9 (7.8)	6 (8.7)	7 (10.8)	
**Active smoking**	13 (5.2)	10 (8.6)	0 (0.0)	3 (4.6)	0.037
**Comorbidities**					
**Cerebrovascular accident**	88 (35.2)	25 (21.6)	37 (53.6)	26 (40)	< 0.001
**Alzheimer's disease**	32 (12.8)	13 (11.2)	14 (20.3)	5 (7.7)	0.072
**Parkinson's disease**	23 (9.2)	9 (7.8)	12 (17.4)	2 (3.1)	0.013
**Epilepsy**	21 (8.4)	3 (2.6)	15 (21.7)	3 (4.6)	< 0.001
**Ischemic heart disease**	47 (18.8)	23 (19.8)	12 (17.4)	12 (18.5)	0.916
**Congestive heart failure**	33 (13.2)	9 (7.8)	10 (14.5)	14 (21.5)	0.030
**Cardiomyopathy**	19 (7.6)	5 (4.3)	8 (11.6)	6 (9.2)	0.165
**Hypertension**	184 (73.6)	78 (67.2)	56 (81.2)	50 (76.9)	0.090
**Asthma**	18 (7.2)	9 (7.8)	6 (8.7)	3 (4.6)	0.627
**Chronic lung disease**	45 (18.0)	16 (13.8)	18 (26.1)	11 (16.9)	0.105
**Cirrhosis**	5 (2.0)	1 (0.9)	1 (1.4)	3 (4.6)	0.208
**Type 2 diabetes**	83 (33.2)	30 (25.9)	29 (42.0)	24 (36.9)	0.059
**Chronic kidney disease**	72 (28.8)	27 (23.3)	26 (37.7)	19 (29.2)	0.112
**Dyslipidemia**	144 (57.6)	59 (50.9)	50 (72.5)	35 (53.8)	0.012
**Autoimmune disease**	6 (2.4)	2 (1.7)	3 (4.3)	1 (1.5)	0.461
**History of inhaled corticosteroid use**	27 (10.8)	8 (6.9)	12 (17.4)	7 (10.8)	0.084
**History of insulin use**	20 (8.0)	7 (6.0)	9 (13.0)	4 (6.2)	0.193
**Clinical signs and laboratory findings**					
**Systolic blood pressure (< 90 mmHg)**	27 (10.8)	7 (6.0)	8 (11.6)	12 (18.5)	0.034
Mean ± SD	127.3 ± 27.1	130.9 ± 25.3	124.4 ± 25.7	123.9 ± 30.8	0.144
Median (min–max)	128.0 (56.0–193.0)	130.0 (82.0–193.0)	128.0 (56.0–181.0)	127.0 (66.0–183.0)	
**Diastolic blood pressure (< 60 mmHg)**	63 (25.2)	19 (16.4)	21 (30.4)	23 (35.4)	0.009
Mean ± SD	72.6 ± 16.2	74.6 ± 15.2	70.5 ± 16.2	70.7 ± 17.5	0.150
Median (min–max)	71.0 (33.0–114.0)	73.5 (40.0–114.0)	68.0 (33.0–110.0)	70 (39.0–110.0)	
**Mean arterial pressure (< 65 mmHg)**	20 (8.0)	113 (97.4)	63 (91.3)	54 (83.1)	0.003
Mean ± SD	90.7 ± 17.8	93.3 ± 16.1	88.4 ± 17.3	88.4 ± 20.6	0.094
Median (min–max)	92.2 (41.0–140.0)	93.2 (56.0–140.3)	86.3 (40.7–123.3)	92.0 (51.7–131.7)	
**Pulse pressure (mmHg)**					
Mean ± SD	54.9 ± 21.3	56.3 ± 22.2	53.9 ± 20.8	53.2 ± 20.5	0.586
Median (min–max)	50.0 (10.0–127.0)	51.5 (10.0–127.0)	53.0 (15.0–109.0)	50.0 (10.0–106.0)	
**Pulse rate (≥ 120 bpm)**	44 (17.6)	23 (19.8)	18 (26.1)	14 (21.5)	
Mean ± SD	103.4 ± 22.2	101.8 ± 23.5	105.7 ± 20.9	103.9 ± 21.1	0.518
Median (min–max)	100.0 (60.0–200.0)	98.0 (61.0–200.0)	104 (60.0–160.0)	102.0 (60.0–150.0)	
**Respiratory rate (> 30/min)**	44 (17.6)	17 (14.7)	12 (17.4)	15 (23.1)	0.361
**Body temperature (°C)**					0.035
< 36.0	5 (2.0)	2 (1.7)	2 (2.9)	1 (1.5)	
36.0–37.7	106 (42.4)	51 (44.0)	37 (53.6)	18 (27.7)	
≥ 37.8	139 (55.6)	63 (54.3)	30 (43.5)	46 (70.8)	
**Alteration of consciousness**	100 (40.0)	34 (29.3)	38 (55.1)	28 (43.1)	0.002
**Hemoglobin (≥ 10 g/dL)**	174 (69.6)	94 (81.0)	41 (59.4)	39 (60.0)	0.001
**Sodium (≥ 130 mEq/L)**	230 (92.0)	109 (94.0)	63 (91.3)	58 (89.2)	0.514
**Bicarbonate (≤ 15 mEq/L)**	9 (3.6)	3 (2.6)	4 (5.8)	2 (3.1)	0.508
**Blood urea nitrogen (> 20 mg/dL)**	137 (54.8)	49 (42.2)	46 (66.7)	42 (64.6)	0.001
**Systemic inflammatory response syndrome**	199 (79.6)	83 (71.5)	57 (82.6)	59 (90.8)	0.007
**qSOFA**					< 0.001
0	56 (22.4)	41 (35.3)	9 (13.0)	6 (9.2)	
1	99 (39.6)	44 (37.9)	28 (40.6)	27 (41.5)	
2	76 (30.4)	27 (23.3)	25 (36.2)	24 (36.9)	
3	19 (7.6)	4 (3.4)	7 (10.1)	8 (12.3)	
**Radiological findings**					< 0.001
Alveolar infiltration	150 (60.0)	51 (44.0)	46 (66.7)	53 (81.5)	
Interstitial infiltration	80 (32.0)	58 (50.0)	15 (21.7)	7 (10.8)	
Lobar pneumonia	4 (1.6)	2 (1.7)	2 (2.9)	0 (0.0)	
Multilobar pneumonia	9 (3.6)	5 (4.3)	3 (4.3)	1 (1.5)	
Pleural effusion	5 (2.0)	0 (0.0)	3 (4.3)	2 (3.1)	
Acute respiratory distress syndrome	2 (0.8)	0 (0.0)	0 (0.0)	2 (3.1)	
**Process of care**					
**Using invasive mechanical ventilation**	135 (54.0)	56 (48.3)	36 (52.2)	43 (66.2)	0.064
**Vasopressor medication use**	57 (22.8)	20 (17.2)	15 (21.7)	22 (33.8)	0.037
**Time to empirical antibiotics (hours)**					< 0.001
mean ± SD	6.2 ± 9.8	5.1 ± 8.4	5.8 ± 7.7	8.4 ± 13.2	0.088
median (min–max)	3.0 (0.3–96.0)	2.0 (0.3–52.0)	4.0 (0.5–40.0)	5.0 (0.3–96.0)	
< 1	62 (24.8)	43 (37.1)	13 (18.8)	6 (9.2)	
1–3	75 (30.0)	40 (34.5)	19 (27.5)	16 (24.6)	
≥ 3	113 (45.2)	33 (28.4)	37 (53.6)	43 (66.2)	
**Intensive care unit admission**	69 (27.6)	34 (29.3)	8 (11.6)	27 (41.5)	< 0.001

CAP; community-acquired pneumonia, HCAP; healthcare-associated pneumonia, HAP; hospital-acquired pneumonia, qSOFA; quick sequential organ failure assessment, SD; standard deviation.

### Bacterial etiology in CAP, HCAP and HAP

The microbiologic etiology of pneumonia is presented in Table 2. In all, 177 (70.8%) participants submitted sputum cultures, and 118 (66.7%) of those tested positive. The present study reported that overall pneumonia caused by gram-negative pathogens accounted for 60.5%. *P. aeruginosa* was a frequent gram-negative pathogen among these participants, especially for patients with HCAP and HAP. *P. aeruginosa* was the causative agent in 10.9% in CAP, 26.3% in HCAP and 26.8% in HAP (*p*-value CAP vs. HCAP and CAP vs. HAP < 0.05). Additionally, we found that 49.2% of patients with pneumonia submitting sputum cultures were caused by drug-resistant pathogens. Methicillin-resistant *Staphylococcus aureus* was found in CAP, HCAP and HAP accounting for 1.6, 3.5 and 12.5%, respectively (*p*-value CAP vs. HAP < 0.05). *Klebsiella pneumoniae* ESBL/MDR was the causative agent in CAP, HAP and HCAP comprising 0, 7.0 and 10.7%, respectively (*p*-value CAP vs. HCAP and CAP vs. HAP < 0.05). No difference was found in the proportion of *Escherichia coli* ESBL/MDR found in CAP, HAP and HCAP. *Acinetobacter baumannii* MDR was found at 0, 10.5 and 17.9% in CAP, HCAP and HAP, respectively (*p*-value CAP vs. HCAP and CAP vs HAP < 0.05).

**Table 2:** Microbiological etiology.

Pathogen	n (%)	CAP (n = 64)	HCAP (n = 57)	HAP (n = 56)
**Sputum culture (n = 177)**				
Not performed	73 (29.2)	52 (44.8)	12 (17.4)	9 (13.8)
Negative	59 (23.6)	33 (28,4)	18 (26.1)	8 (12.3)
Positive	118 (47.2)	31 (26.7)	39 (56.5)	48 (73.8)
***Staphylococcus auresus (MSSA)***	13 (7.3)	5 (7.8)	2 (3.5)	6 (10.7)
***Staphylococcus auresus (MRSA) †***	10 (5.6)	1 (1.6)	2 (3.5)	7 (12.5)
***Streptococcus pneumoniae ¶***	8 (4.5)	4 (6.3)	4 (7.0)	0 (0.0)
***Escherichia coli ¶***	7 (4.0)	3 (4.7)	4 (7.0)	0 (0.0)
***Escherichia coli ESBL/MDR***	13 (7.3)	2 (3.1)	6 (10.5)	5 (8.9)
***Klebsiella pneumoniae***	25 (14.1)	10 (15.6)	6 (10.5)	9 (16.1)
Klebsiella pneumoniae ESBL/MDR *, †	10 (5.6)	0 (0.0)	4 (7.0)	6 (10.7)
***Klebsiella pneumoniae CRE***	2 (1.1)	0 (0.0)	0 (0.0)	2 (3.6)
***Pseudomonas spp. †***	16 (9.0)	2 (3.1)	6 (10.5)	8 (14.3)
Pseudomonas aeruginosa *, †	37 (20.9)	7 (10.9)	15 (26.3)	15 (26.8)
***Acinetobacter baumannii***	6 (3.4)	2 (3.1)	2 (3.5)	2 (3.6)
Acinetobacter baumannii MDR *, †	16 (9.0)	0 (0.0)	6 (10.5)	10 (17.9)
***Proteus mirabilis***	5 (2.8)	0 (0.0)	3 (5.3)	2 (3.6)
***Haemophilus influenzae***	5 (2.8)	4 (6.1)	0 (0.0)	1 (1.8)
***Stenotrophomonas maltophilia***	8 (4.5)	1 (1.6)	2 (3.5)	5 (8.9)
***Mycoplasma pneumoniae (n* = *6)***	3 (50.0)	3 (60.0)	0 (0.0)	0 (0.0)
***Gram positive pathogens***	29 (16.4)	10 (15.6)	6 (10.5)	13 (23.2)
Gram negative pathogens *,†,¶	107 (60.5)	24 (37.5)	37 (64.9)	46 (82.1)
Drug resistant pathogens *, †,¶	87 (49.2)	15 (23.4)	31 (54.4)	41 (73.2)
Influenza virus (n = 139) *				
Influenza A virus	51 (36.7)	43 (42.2)	6 (20.0)	2 (28.6)
Influenza B virus	14 (10.1)	13 (12.7)	1 (3.3)	0 (0.0)
**Other respiratory viruses (n = 29)**	8 (27.6)	6 (28.6)	1 (16.7)	1 (50.0)

CAP; community acquired pneumonia, HCAP; healthcare-associated pneumonia, HAP; hospital-acquired pneumonia, qSOFA; quick sequential organ failure assessment, SD; standard deviation.

Gram positive pathogens included MSSA, MRSA, *Streptococcus spp.*

Gram negative pathogens included *Escherichia spp., Klebsiella spp., Pseudomonas spp. Acinetobacter spp., Proteus mirabilis, Haemophilus influenzae and Stenotrophomonas maltophilia.*

Atypical pathogens included *Mycoplasma spp.*

Drug resistance pathogens included MRSA, *Escherichia coli ESBL/MDR, Klebsiella pneumoniae ESBL/MDR, Klebsiella pneumoniae CRE, Pseudomonas aeruginosa, Acinetobacter baumannii, Acinetobacter baumannii MDR, Proteus mirabilis.*

Other respiratory viruses included Adenovirus, Rhinovirus, Metapneumovirus, Parainfluenza virus, Respiratory syncytial virus.

*p < 0.05 (CAP vs HCAP), ^†^*p* < 0.05 (CAP vs HAP), ^¶^*p* < 0.05 (HCAP vs HAP).

### Mortality rate among inpatients with pneumonia

**Figure 1:**
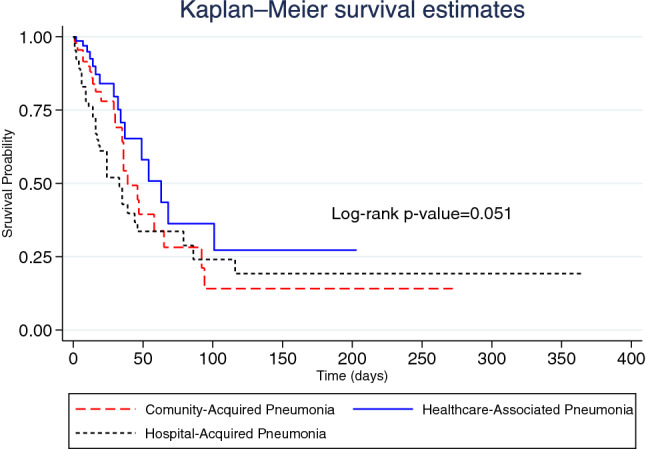
Kaplan–Meier graph of inhospital mortality by pneumonia category.

In terms of inhospital mortality, the overall median survival time was 44.0 days (95% CI 34.7–53.3). The median survival time of patients with CAP, HCAP and HAP was 39.0 days (95% CI 28.0–50.0), 63.0 days (95% CI 40.2–85.8) and 33.0 days (95% CI 20.7–45.3), respectively. Figure 1 illustrates the Kaplan–Meier graph of inhospital mortality by pneumonia category (*p*-value = 0.051). A total of 78 (31.2%) inpatients died in hospital, representing an inhospital mortality rate of 1.25 (95% CI 0.99–1.56) per 100 person-days. Among patients with CAP, the inhospital mortality rate was 1.29 (95% CI 0.84–1.89) per 100 person-days and 0.88 (95% CI 0.50–1.43) per 100 person-days among those with HCAP. The inhospital mortality rate among patients with HAP was 1.48 (95% CI 1.04–2.05) per 100 person-days.

**Figure 2:**
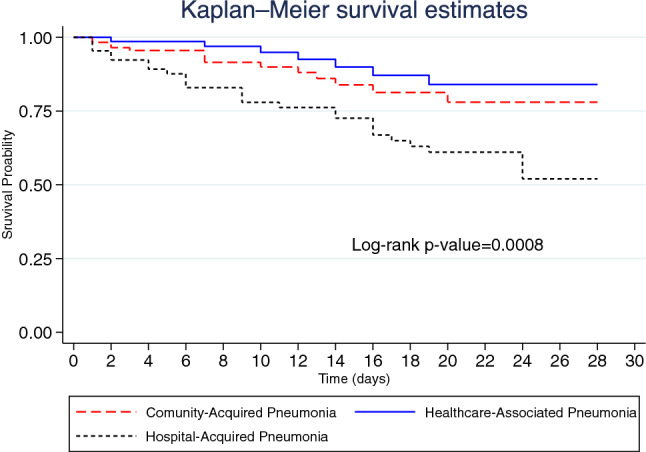
Kaplan–Meier graph of 28-day mortality by pneumonia category.

Figure 2 illustrates the Kaplan–Meier graph of 28-day mortality by pneumonia category (*p*-value = 0.0008). A total of 48 (19.2%) inpatients died within 28 days, indicating a 28-day mortality rate of 1.33 (95% CI 0.98–1.77) per 100 person-days. Among patients with CAP, the 28-day mortality rate was 1.04 (95% CI 0.57–1.74) per 100 person-days and 0.62 (95% CI 0.25–1.28) per 100 person-days among those with HCAP. The 28-day mortality rate among patients with HAP was 2.40 (95% CI 1.58–3.49) per 100 person-days.

### Risk factors for mortality among inpatients with pneumonia

Univariable cox regression analysis for risk factors of inhospital mortality and 28-day mortality was performed and is presented in Tables 3 and 4, respectively. Table 5 presents independent risk factors for mortality among patients with pneumonia.

**Table 3:** Univariable cox regression analysis for risk factors of inhospital mortality.

Risk factors	Total N	No. of death	Person-day of observation	Mortality rate per 100 person-days	Crude Hazard Ratio (95% CI)	*p*-value
**Total**	250	78	6249	1.25		
**Pneumonia**						
CAP	116	26	2011	1.29	1	
HCAP	69	16	1811	0.88	0.68 (0.36–1.27)	0.228
HAP	65	36	2427	1.48	1.38 (0.83–2.31)	0.213
**Female**	104	31	2069	1.50	1.21 (0.76–1.90)	0.425
**Age (years)**	250	83	6249	1.25	1.03 (1.01–1.06)	0.006
< 65	50	3	958	0.30	1	
65–74	40	7	836	0.80	2.38 (0.62–9.24)	0.209
≥ 75	160	68	4455	1.50	3.87 (1.21–12.33)	0.022
**Marital status**						
Married	174	53	4428	1.20	1	
Single	31	4	427	0.94	0.67 (0.24–1.87)	0.449
Widowed/divorced	45	21	1394	1.51	1.16 (0.70–1.92)	0.574
**Active smoking**	13	3	102	2.94	2.30 (0.71–7.46)	0.165
**Bed ridden**	87	29	2989	0.97	0.63 (0.40–1.01)	0.056
**Comorbidities**						
**Cardiomyopathy**	19	8	233	3.43	2.72 (1.28–5.77)	0.009
**Chronic kidney disease**	72	29	1591	1.82	1.68 (1.06–2.67)	0.028
**History of insulin use**	20	10	450	2.22	1.69 (0.87–3.29)	0.125
**Clinical signs and laboratory findings**						
**Mean arterial pressure (< 65 mmHg)**	20	65	5740	1.13	1.92 (1.06–3.49)	0.032
**Respiratory rate (> 30/min)**	44	21	973	2.16	1.82 (1.10–3.02)	0.020
**Body temperature (°C)**	250	78	6249	1.25	0.95 (0.78–1.15)	0.594
< 36.0	106	28	2705	1.04	4.52 (1.35–15.14)	0.015
36.0–37.8	5	3	56	5.36	1	
≥ 37.8	139	47	3488	1.35	1.24 (0.78–1.99)	0.366
**Bicarbonate (≤ 15 mEq/L)**	9	6	97	6.19	5.13 (2.16–12.19)	< 0.001
**Blood urea nitrogen (> 20 mg/dL)**	137	66	3579	1.84	3.58 (1.93–6.63)	< 0.001
**Systemic inflammatory response syndrome**	199	70	5134	1.36	1.86 (0.89–3.87)	0.097
**qSOFA ≥ 2**	95	48	2534	1.89	2.22 (1.41–3.51)	< 0.001
**Process of care**						
**Using invasive mechanical ventilation**	135	71	4566	1.55	3.77 (1.71–8.31)	< 0.001
**Intensive care unit admission**	69	43	2059	2.09	2.41 (1.54–3.77)	< 0.001

CAP; community-acquired pneumonia, HCAP; healthcare-associated pneumonia, HAP; hospital-acquired pneumonia, qSOFA; quick sequential organ failure assessment.

**Table 4:** Univariable cox regression analysis for risk factors of 28-day mortality.

Risk factors	Total N	No. of death	Person-day of observation	Mortality rate per 100 person-days	Crude Hazard Ratio (95% CI)	*p*-value
**Total**	250	48	3598	1.33		
**Pneumonia**						
CAP	116	14	1348	1.04	1	
HCAP	69	7	1124	0.62	0.62 (0.25–1.53)	0.297
HAP	65	27	1126	2.40	2.31 (1.20–4.43)	0.012
**Female**	104	22	1347	1.63	1.42 (0.80–2.51)	0.232
**Age (years)**	250	48	3598	1.33	1.02 (1.00–1.05)	0.075
< 65	50	3	486	0.62	1	
65–74	40	6	516	1.16	1.97 (0.49–7.88)	0.339
≥ 75	160	39	2596	1.50	2.53 (0.78–8.20)	0.123
**Marital status**						
Married	174	53	4428	1.20	1	
Single	31	4	427	0.94	0.67 (0.24–1.87)	0.449
Widowed/divorced	45	21	1394	1.51	1.16 (0.70–1.92)	0.574
**Active smoking**	13	3	98	3.06	2.45 (0.75–7.98)	0.136
**Bed ridden**	87	11	1518	0.72	0.42 (0.21–0.83)	0.012
**Comorbidities**						
**Cardiomyopathy**	19	6	222	2.70	2.15 (0.91–5.08)	0.080
**Chronic kidney disease**	72	17	1054	1.61	1.35 (0.74–2.43)	0.327
**History of insulin use**	20	8	282	2.84	2.35 (1.10–5.01)	0.028
**Clinical signs and laboratory findings**						
**Mean arterial pressure (< 65 mmHg)**	20	8	328	2.44	2.03 (0.95–4.33)	0.068
**Respiratory rate (> 30/min)**	44	19	663	2.87	2.74 (1.16–6.47)	< 0.001
**Body temperature (°C)**	250	48	3598	1.33	0.89 (0.69–1.14)	0.346
< 36.0	106	3	3	5.36	5.10 (1.47–17.70)	0.010
36.0–37.8	5	15	1524	0.98	1	
≥ 37.8	139	30	2018	1.49	1.50 (0.81–2.79)	0.200
**Bicarbonate (≤ 15 mEq/L)**	9	6	97	6.19	5.13 (2.16–12.19)	< 0.001
**Blood urea nitrogen** **(> 20 mg/dL)**	137	40	2154	1.86	3.39 (1.58–7.24)	0.002
**Systemic inflammatory response syndrome**	199	45	2953	1.52	3.31 (1.03–10.64)	0.045
**qSOFA ≥ 2**	95	31	1522	2.03	2.52 (1.39–4.55)	0.002
**Process of care**						
**Using invasive mechanical ventilation**	135	41	2393	1.71	3.06 (1.36–6.87)	0.007
**Intensive care unit admission**	69	29	1099	2.64	3.46 (1.94–6.17)	< 0.001

CAP: community-acquired pneumonia, HCAP; healthcare-associated pneumonia, HAP; hospital-acquired pneumonia, qSOFA; quick sequential organ failure assessment.

**Table 5:** Multivariable cox regression analysis for risk factors of inhospital mortality and 28-day mortality.

Risk factors	Inhospital mortality		28-day mortality	
	Adjusted HR (95% CI)	*p*-value	Adjusted HR (95% CI)	*p*-value
**Pneumonia**				
HCAP versus CAP	0.60 (0.30–1.20)	0.148	0.54 (0.20–1.45)	0.220
HAP versus CAP	1.75 (1.01–3.03)	0.046	2.81 (1.38–5.75)	0.005
**Female**	1.62 (0.93–2.82)	0.089	2.14 (1.08–4.24)	0.029
**Age (years)**				
65–74 versus < 65	5.39 (1.10–26.3)	0.037	7.31 (1.34–39.94)	0.022
≥ 75 versus < 65	8.99 (2.11–38.22)	0.003	8.97 (1.90–42.38)	0.006
**Marital status**				
Married versus single	0.74 (0.25–2.20)	0.590	0.56 (0.16–1.99)	0.373
Widowed/divorced versus single	0.64 (0.33–1.22)	0.172	0.60 (0.26–1.40)	0.239
**Cardiomyopathy**	2.64 (1.09–6.41)	0.032	2.02 (0.73–5.62)	0.176
**Chronic kidney disease**	1.30 (0.76–2.22)	0.344	1.00 (0.49–2.02)	0.990
**Insulin used**	2.34 (1.11–4.95)	0.026	2.86 (1.19–6.89)	0.019
**Active smoking**	13.89 (3.23–59.62)	< 0.001	14.94 (3.12–71.44)	0.001
**qSOFA ≥ 2**	1.94 (1.17–3.21)	0.010	2.23 (1.15–4.31)	0.018
**Serum bicarbonate ≤ 15 mEq/L**	5.18 (1.97–13.58)	0.001	6.43 (2.31–17.86)	< 0.001
**Using invasive mechanical ventilator**	2.09 (0.90–4.83)	0.085	1.85 (0.78–4.36)	0.162

CAP; community-acquired pneumonia, HCAP; healthcare-associated pneumonia, HAP; hospital-acquired pneumonia, qSOFA; quick sequential organ failure assessment.

After adjusting for potential confounders, the risk factors of inhospital mortality were HAP (AHR 1.75; 95% CI 1.01–3.03) compared with CAP, higher age, cardiomyopathy, history of insulin, active smoking, qSOFA ≥ 2 and serum HCO_3_ ≤ 15 mEq/L. The independent risk factors for 28-day mortality were HAP (AHR 2.81; 95% CI 1.38–5.75) compared with CAP, being female, higher age, history of insulin use, active smoking, qSOFA ≥ 2 and serum HCO_3_ ≤ 15 mEq/L.

## Discussion

To our knowledge, this constitutes a primal report demonstrating a comparison of bacterial etiology and mortality rate among inpatients with CAP, HCAP and HAP in Thailand. We successfully included 250 inpatients with pneumonia in the present study. At baseline, we observed that most study participants were elderly patients with various comorbidities. Our study found that the prevalence of cerebrovascular events, Parkinson’s disease (PD), and epilepsy among patients with HCAP were relatively high compared with those among patients with CAP or HAP. Similarly, a related study reported a high incidence of pneumonia among patients with PD. These patients are well-known to experience dysphagia, leading to either micro-aspiration or aspiration^20–23^. Therefore, early recognition and prompt management of these health conditions among older people may attenuate the risk of hospitalization with pneumonia, and thus, the burden of the disease^24^.

We reported that the most common pathogen of pneumonia in this study was gram-negative P. *aeruginosa,* especially in HCAP and HAP while *Klebsiella pneumonia* was the most common pathogen in CAP. This finding was comparable with that of a related study in a tertiary referral hospital, northern Thailand in 2019 indicating that *P. aeruginosa* was a frequent causative agent in HAP approximating 13.8%^25^. However, another study in Bangkok in 2010 reported that *A. baumannii* was the most common pathogen among inpatients with HAP while *P. aeruginosa* was the second most common cause^26^. In addition, a systematic review of the burden of healthcare-associated infections (HAI) in Southeast Asia illustrated that the most common cause of overall HAI was *P. aeruginosa*^27^*.* Our finding reported that CAP was frequently caused by *Klebsiella pneumonia* which was compatible with the finding of a related study conducted at the Bamrasnaradura Infectious Diseases Institute in central Thailand^28^. However, a recent study in northeast Thailand reported that *S. pneumoniae* was the most common pathogen, identified in 11.4% of inpatients with CAP^29^. Furthermore, reports in the US, Spain and Asia indicated that *S. pneumoniae* was the most common bacterial etiology of adult CAP^30–32^. Our study presented that a half of inpatients with pneumonia was caused by drug-resistant pathogens, i.e., *P. aeruginosa* and *A. baumannii,* respectively. This finding was consistent with a related study in northern Thailand^25^; however, another study conducted in 12 tertiary care hospitals in Thailand reported that *A. baumannii* was the most common drug-resistant pathogen among inpatients with HAP^20,27^. Additionally, the data from nationwide surveillance in Thailand also demonstrated that *A. baumannii complex* was the most common drug-resistant, gram-negative organism causing respiratory infection^10^. Our study suggested the potential tailoring of directed empirical antibiotic regimens and more rapid identification of pathogens and resistance patterns. While implementing antimicrobial stewardship programs and infection control measures such as hand hygiene, contact precautions were suggested to reduce the nosocomial infection of resistant bacterial strains^34–36^.

Our study reported that the overall inhospital mortality rate of inpatients with pneumonia was 31.2% being higher when compared with that in nationwide hospital admission data in 2010 reporting that the mortality rate among adults aged ≥ 60 years with pneumonia was approximately 9.2–15.5%^37^. We found that the inhospital and 28-day mortality rate in CAP and HCAP were comparable while approximately twice higher in HAP. Our study revealed that the inhospital mortality rate in CAP was 22.4% (1.29 deaths/100 person-days) which was relatively high, compared with that in other studies conducted at the Bamrasnaradura Infectious Diseases Institute in Thailand reporting 17.9%^28^. Mortality rates for patients hospitalized with CAP reported by related studies have ranged from 12 to 30%^38–40^. Twenty-eight-day mortality in CAP during our study was 12.1% and tended to be higher than that in Korean tertiary teaching hospitals reporting 6.3%; conversely, the 28-day mortality rate in HCAP in our study was relatively low^41^. Our finding illustrated that inhospital mortality in HAP was 55.4% (1.48 death/100 person-days) which was greater than that in related studies both in Thailand and overseas^20,33^. However, the 28-day mortality rate in HAP in the present study was comparable with those of other related studies ranging from 28.7 to 44.4%^25,26,42^. Therefore, vaccinations for some specific pathogens such as influenza, *S. pneumoniae is* a major preventive strategy for pneumonia acquired in a community setting and plays an essential role in reducing incidence and mortality ^43–45^. In addition, minimizing hospital length of stay is an effective method to lower HAP incidence^46^.

In our study, mortality rates in HAP were worse than those in CAP and HCAP. Using multivariable cox regression analysis, HAP was a significant risk factor for inhospital and 28-day mortality. This was possible because disease severity itself is related to mortality and may result from the high prevalence of drug-resistant pathogens in HAP^34,35^.

Several studies in Thailand reported that male patients with pneumonia had higher mortality rates than females^25,28^. In this study, female patients with CAP, HCAP and HAP accounted for 52.6, 31.9 and 32.3%, respectively. After adjusting for potential confounders, females were significantly associated with 28-day mortality (AHR 2.14). However, the related study in Korea indicated that sex was not associated with mortality among inpatients with pneumonia^41^. The present study reported that a dose–response relationship existed between higher age and mortality rates among inpatients with pneumonia. Similarly, a nationwide hospital data in 2010 and the study conducted at the Bamrasnaradura Infectious Diseases Institute in Thailand reported that inpatients with pneumonia who were higher aged tended to increase in mortality^22,28^. This finding could be explained by the decline of immune function and anatomic and functional changes^49–51^. Nevertheless, some studies reported no difference in age between survivors and nonsurvivors^19,32^.

We found that cardiomyopathy was an independent risk for inhospital mortality. Correspondingly, nationwide hospital admission data in 2010 indicated that the odds of death among inpatients having a history of heart disease was 2.47 times (95% CI 2.38–2.56) the odds of death among those without heart disease ^37^. Patients with cardiomyopathy were prone to have ventricular tachycardia and ventricular fibrillation, which were crucial causes of death^52,53^. Among patients with pneumonia, systemic infections rapidly aggravated cytokine-mediated, ventricular electrical remodeling and worsened their heart condition as cardiogenic shock, consequently increasing mortality^54,55^.

Our study demonstrated that inpatients using insulin had higher inhospital and 28-day mortality rates when compared with those not using insulin. Certainly, insulin is prescribed for patients with type 2 diabetes (T2D) with poor glycemic control, having a long duration with T2D and being prone to microvascular and macrovascular complications^56–58^. Furthermore, hyperglycemia causes dysfunction of the immune response, diminishing control of the spread of invading pathogens leading to morbidity and mortality^59^.

The existing evidence indicated that smoking caused respiratory symptoms, bronchial hyperresponsiveness and excessive decline in lung function^60^. The present study reported that inpatients with pneumonia who were active smokers had a very high risk for death during admission in hospitals (AHR 13.9; 95% CI 3.2–59.6) and within 28 days (AHR 14.9; 95% CI 3.1–71.4). This finding was compatible with a related study in Spain demonstrating that current smoking was an independent risk factor (AOR 5.0; 95% CI 1.8–13.5) for 30-day mortality. Moreover, current smokers with pneumonia frequently develop severe sepsis and worsening outcomes^61^.

We found that inpatients with pneumonia having serum bicarbonate at baseline $\le$ 15 mEq/L tended to have inhospital and 28-day mortality. Similarly, a related report indicated that critically ill patients with metabolic acidosis had higher mortality, compared with those with no metabolic acidosis^62^. Acid–base disturbances will decrease cardiopulmonary function, especially metabolic acidosis, which interferes with contractility by involving the binding and release of calcium to the sarcoplasmic reticulum in the cardiocyte^50^. Therefore, acid-based disturbance among patients admitted in the hospital should be assessed and appropriately corrected to attenuate the mortality rate.

According to the Third International Consensus Definitions for Sepsis and Septic Shock, qSOFA criteria was be used to prompt clinicians to further investigate organ dysfunction and to initiate or escalate therapy as appropriate^18^. The present study reported that the inhospital and 28-day mortality among inpatients with pneumonia presenting qSOFA scores of 2 or greater were significantly higher, compared with those presenting a qSOFA scores of less than 2. The qSOFA did not require laboratory tests and could be assessed quickly and repeatedly^18^; therefore, it could help inform appropriate management and improve patient outcomes.

The present study, a retrospective cohort study, included inpatients before the beginning of the coronavirus disease 2019 (COVID-19) pandemic. At present, COVID-19 may significantly facilitate death among people, especially adults aged ≥ 60 years and among people with severe underlying health conditions^64^. Furthermore, a recent report indicated that secondary bacterial infection among patients with COVID-19 is a stronger predictor of death than among patients with influenza^65^. Because the clinical presentations of COVID-19 may be challenging to distinguish from bacterial pneumonia, careful surveillance and prompt, empirical antimicrobial treatment for bacterial pneumonia (CAP, HAP), based on local bacterial epidemiology, may also be reasonable among patients with COVID-19^65^.

Some limitations were encountered in this study. First, this constituted a hospital-based retrospective cohort study in a single university hospital in Bangkok, Thailand. Hence, it may represent epidemiologic data and mortality rates of pneumonia in medical centers. In addition, we excluded immunocompromised individuals; thus, the generalizability of the results should be considered. Second, the sputum culture was not performed among all patients because sputum samples from patients who did not intubate may exhibit low quality and are easy to contaminate. Third, multiplex PCR for respiratory pathogens is a high-cost investigation in our resource-limited country. Therefore, using the empirical therapy strategy to determine atypical pathogens instead of PCR investigation is routinely practiced in Thailand. In consequence, the prevalence of atypical pathogens and respiratory viruses in CAP were underestimated in the present study because only a few patients received specific investigations including multiplex PCR for respiratory pathogens and specific antibodies for atypical pathogens. Finally, the study used a small sample size; therefore, the association between well-known risk factors such as required mechanical ventilator and chronic kidney disease and outcomes could be not presented.

## Conclusion

In conclusion, our study emphasized that gram-negative pathogens were a common cause of CAP, HCAP and HAP. Drug-resistant pathogens particularly *P. aeruginosa* played a major contributing cause among patients with HCAP and HAP. Hence, empirical therapy to determine gram negative pathogens should be a major consideration. The mortality of pneumonia remains high, especially in higher aged groups and among inpatients with HAP. Individual risks factors including cardiomyopathy, active-smoker and insulin use posed potential risks for mortality. Initial qSOFA and acid-based disturbance should be assessed to provide further appropriate management and improve clinical outcomes.

## Data Availability

The datasets generated or analyzed during the current study are not publicly available because they contain privacy information. Thus, due to ethics restrictions and concerns, the datasets are available from the corresponding author upon reasonable request.

## Abbreviations

CAP: Community Acquired Pneumonia
HCAP: Healthcare Associated Pneumonia
HAP: Hospital Acquired Pneumonia
HAI: Healthcare Associated Infections
CURB-65: New onset Confusion, blood urea nitrogen > 7 mmol/L (19 mg/dL), respiratory rate ≥ 30/minute, systolic blood pressure < 90 mmHg or diastolic blood pressure ≤ 60 mmHg and age ≥ 65 years
AHR: Adjusted Hazard Ratio
95% CI: 95% Confidence Interval
SIRS: Systemic Inflammatory Response Syndrome
qSOFA: Quick Sequential Organ Failure Assessment
PD: Parkinson’s Disease
COVID-19: Coronavirus Disease 2019
